# Longitudinal corpus callosum microstructural decline in early-stage Parkinson’s disease in association with akinetic-rigid symptom severity

**DOI:** 10.1038/s41531-022-00372-1

**Published:** 2022-08-29

**Authors:** Matthew Amandola, Agniva Sinha, Mark J. Amandola, Hoi-Chung Leung

**Affiliations:** 1 Department of Psychology, Integrative Neuroscience Program, Stony Brook University, Stony Brook, NY USA; 2 Renaissance School of Medicine, Stony Brook University, Stony Brook, NY USA

**Keywords:** Parkinson's disease, Parkinson's disease, Predictive markers

## Abstract

Previous diffusion tensor imaging (DTI) studies of Parkinson’s disease (PD) show reduced microstructural integrity of the corpus callosum (CC) relative to controls, although the characteristics of such callosal degradation remain poorly understood. Here, we utilized a longitudinal approach to identify microstructural decline in the entire volume of the CC and its functional subdivisions over 2 years and related the callosal changes to motor symptoms in early-stage PD. The study sample included 61 PD subjects (*N* = 61, aged 45–82, 38 M & 23 F, H&Y ≤ 2) from the Parkinson’s Progressive Markers Initiative database (PPMI). Whole-brain voxel-wise results revealed significant fractional anisotropy (FA) and mean diffusivity (MD) changes in the CC, especially in the genu and splenium. Using individually drawn CC regions of interest (ROI), our analysis further revealed that almost all subdivisions of the CC show significant decline in FA to certain extents over the two-year timeframe. Additionally, FA seemed lower in the right hemisphere of the CC at both time-points, and callosal FA decline was associated with FA and MD decline in widespread cortical and subcortical areas. Notably, multiple regression analysis revealed that across-subject akinetic-rigid severity was negatively associated with callosal FA at baseline and 24 months follow-up, and the effect was strongest in the anterior portion of the CC. These results suggest that callosal microstructure alterations in the anterior CC may serve as a viable biomarker for akinetic-rigid symptomology and disease progression, even in early PD.

## Introduction

Parkinson’s disease (PD) is a neurodegenerative disorder which stems from the progressive loss of dopaminergic neurons in the substantia nigra pars compacta of the midbrain^1^. Patients with PD experience a severe detriment to quality of life and suffer from cognitive, motor, and sensory impairments^2–5^. Past research has shown that PD pathology extends past the midbrain, striatum, and sensorimotor-related circuits and eventually results in widespread neurological alterations in both white matter^6^ and gray matter structures^7^. While volumetric white matter results have been variable in studies of PD^8^, accumulating evidence suggests measurable changes in microstructural integrity in major white matter tracts, such as the corpus callosum (CC), superior and inferior longitudinal fasciculus, uncinate fasciculus, and cingulum^9–11^.

Among the altered white matter structures observed in PD, most consistent disease-related alterations were found in the CC, which is the main commissural fiber bundle that connects the two cerebral hemispheres (see review by van der Knaap & van der Ham, 2011)^12^. The CC comprises four anatomically organized segments: the rostrum, genu, body, and splenium, which are identified by their anterior-posterior position and their pattern of cortical connections^13,14^. Accordingly, the CC can be divided into five different functional segments, defined by diffusion tractography: the prefrontal, premotor, motor, somatosensory, and temporal-parietal-occipital subsections^14^. Recent research has attempted to quantify callosal microstructural degradation in PD and other neurodegenerative disorders by measuring water diffusion in brain tissues using diffusion tensor imaging (DTI) methods^15,16^. DTI measures such as fractional anisotropy (FA) and mean diffusivity (MD) provide noninvasive in-vivo measurements of myelination^17^, dense axon bundling, and overall white matter integrity^18^.

Previous DTI studies have consistently reported reduced callosal microstructural integrity in PD samples using voxel-wise whole-brain analysis, though it is unclear whether such degradation in PD is restricted to particular callosal segments. For example, while the many replicated microstructural changes in PD involve the genu portion of the CC^6,11,19,20^, there are equally many studies that do not report significant genu alterations^21–24^. Similar mixed findings are observed for other callosal segments. One factor contributing to these discrepancies is the prevalence of atlas-based, group-averaging approaches used in whole-brain group analysis. The image normalization procedure together with large inter-subject variability can contribute to both false positives and negatives^25^. To mitigate this issue, some studies automatically segmented the CC (e.g., Wu et al.)^26^, or used an atlas-based normalized regions of interest (ROI) approach (e.g., Theilmann et al.; Rau et al.)^22,27^ to examine callosal degradation in PD. Findings from these callosal ROI studies were also inconsistent and some still treated the CC as a homogeneous unit, because the typical auto-segmentmentation approaches are still subject to partial volume effect. Further, a few studies only examined the mid-sagittal slice of the CC^28^. A more individualized approach to define the full CC in subjects’ native spaces is necessary for studying the intricate characteristics of callosal degradation and relating them to specific motor and cognitive outcomes across patients.

Further, the majority of previous studies are cross-sectional (see review by Atkinson-Clement et al.)^15^, which are susceptible to heterogeneous samples that reduce detection power and do not afford characterization of the time course of microstructural degradation as the disease progresses. In particular, it is unclear whether microstructural abnormalities occur differentially across the CC during early stages of the disease, since the majority of the studies examined subjects past stage II on the Hoehn and Yahr scale^29^. In fact, most previous studies provided cross-sectional data from either more advanced stages of PD^11,19,21,28,30^, or a mixture of stages^22,23^. However, it is well recognized that physiological changes in PD happen well before clinical symptoms are detectable^31^, implying that PD patients in middle and later stages have already sustained many years of microstructural decline. There are few longitudinal studies in PD that examine the CC to this date^26,27^. This leaves a gap in knowledge on the early development of callosal microstructural degradation in PD. A longitudinal DTI study on early-stage PD is necessary to elucidate the time course of microstructural degradation.

As the anatomical pattern and time course of early CC degradation in PD is not known, the current study sought to systematically examine the longitudinal pattern of CC microstructural degradation as PD progresses in early stages by utilizing a larger sample of newly diagnosed PD patients with DTI data at baseline and a 24-month follow-up. Besides the standard whole-brain voxel-wise analysis, we used individually hand-drawn ROIs to study how the CC as a whole and its subdivisions change over time. We also examined the relationship between individual differences in baseline akinetic rigid and tremor symptom severity and DTI measures of CC microstructural integrity across subjects. We also examined the laterality of microstructural degradation in relation to the side of PD motor symptom onset, since the CC is known to have direct connections to ipsilateral corticostriatal neurons^32^. Lastly, we conducted an exploratory whole-brain analysis to examine the potential variation in functional consequences of a degrading CC.

## Results

### Whole-brain voxel-wise analysis of callosal changes across 24 months

**Fig. 1:**
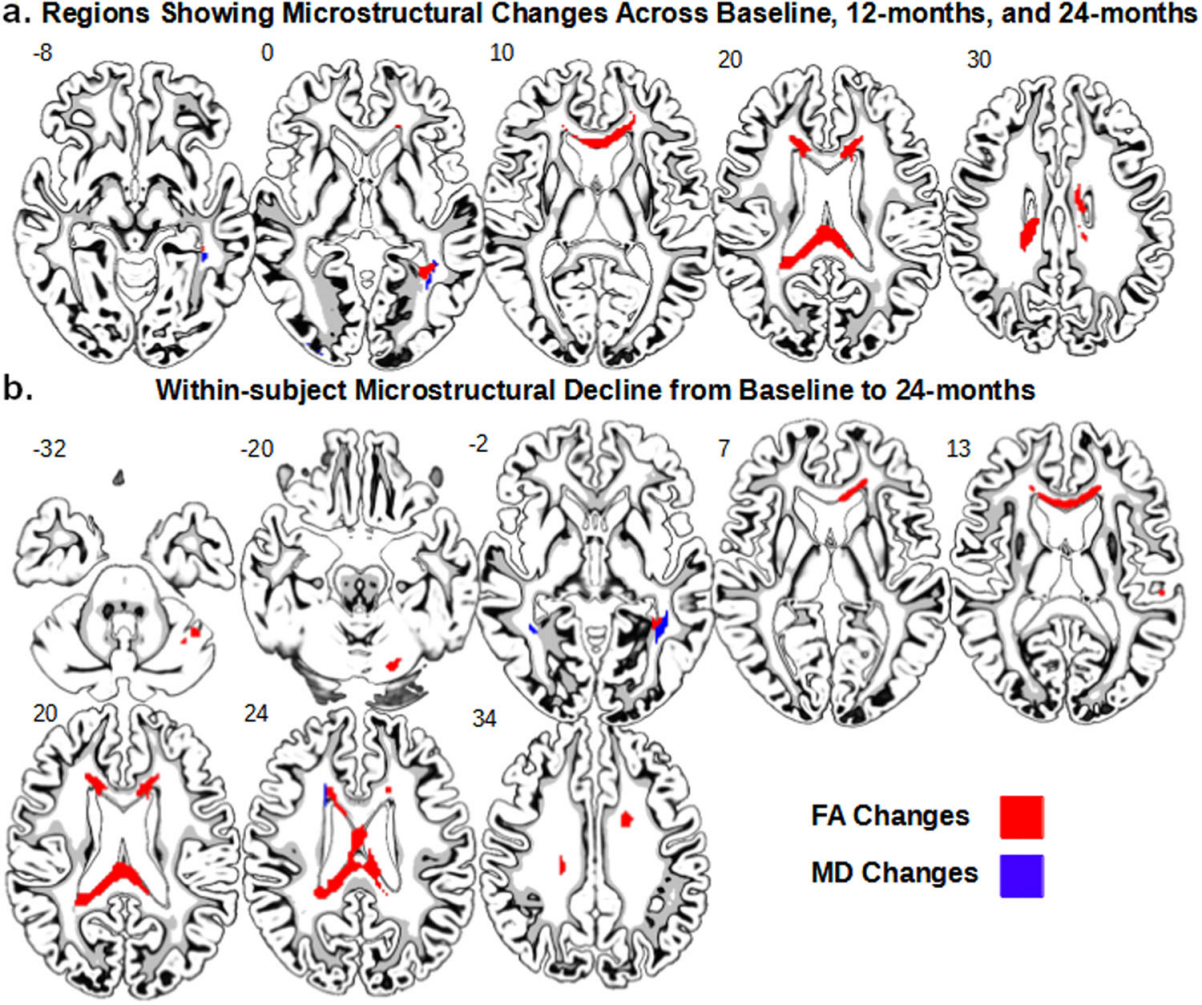
Whole-brain voxel-wise analysis of callosal changes across 24 months. **a** Whole-brain ANOVA data. Suprathreshold clusters show FA (red) and MD (blue) changes across the baseline, 12-month, and 24-month follow-up time-points (FDR *p* < 0.05). **b** Results from paired *t* tests comparing the baseline and 24-month time-points. Suprathreshold clusters show a within-subject microstructural decline (FDR *p* < 0.05). Red indicates areas where FA decreased over time, and blue indicates areas where MD increased over time.

First, in order to replicate past research, we examined whole-brain voxel-wise changes in FA and MD across two years, using the 61 PD subjects with DTI data from all three time-points (baseline, 12-month, and 24-month). Figure 1a shows the voxel clusters that showed significant within-subject changes in microstructural integrity over time (FDR corrected, *p* < 0.05). For FA, there were three main suprathreshold clusters, which were the splenium (*k* = 420), the genu (*k* = 98), and the left posterior thalamic radiation (*k* = 39). For MD, there was one suprathreshold cluster in the left posterior thalamic radiation (*k* = 27). More specifically, paired *t* tests revealed a significant within-subject decline in FA from baseline to 24-month in several areas (see Fig. 1b), including the CC, corona radiata, cerebellum, and thalamic radiation. Full results from the paired *t* tests can be found on Table 1. The opposite effects were also found in FA but not MD in the right anterior corona radiata (*k* = 45) and superior corona radiata (*k* = 42), with greater FA at 24-month in comparison to baseline (data not shown). In sum, significant decreases in FA and increases in MD in multiple parts of the CC were evident longitudinally across a 2-year time period in voxel-wise whole-brain maps.

**Table 1:** Clusters that showed a significant microstructural decline in the paired *t* tests comparing FA/MD at the baseline and 24-month follow-up time-points.

Region	Cluster size (k)	*T* value	*X*	*Y*	*Z*
FA results					
Body & splenium	877	5.61	12	−38	22
Genu	351	5.39	−18	32	12
Left posterior corona radiata	78	5.25	−38	−48	−2
Left cerebellum	35	4.70	−42	−54	−3
	21	4.54	−20	−70	−20
	34	4.53	−46	−46	−36
Left somatosensory white matter	17	4.12	−4	−48	52
Right medial occipital white matter	18	4.04	20	−70	16
Left superior portion of posterior corona radiata	31	3.97	−30	−52	18
Left inferior parietal white matter	14	3.88	−56	−28	14
MD results					
Left posterior thalamic radiation	87	5.06	−40	−48	−2
Left frontal white matter	21	4.55	−36	22	12
Right anterior corona radiata	18	4.10	20	12	24
Right thalamic radiation	18	3.95	36	−52	−2
Right superior occipital white matter	14	4.92	20	−82	48

*k* number of voxels; *X*, *Y*, *Z* = peak coordinates.

### Individually based ROI analysis of callosal changes from baseline to 24 months

**Fig. 2:**
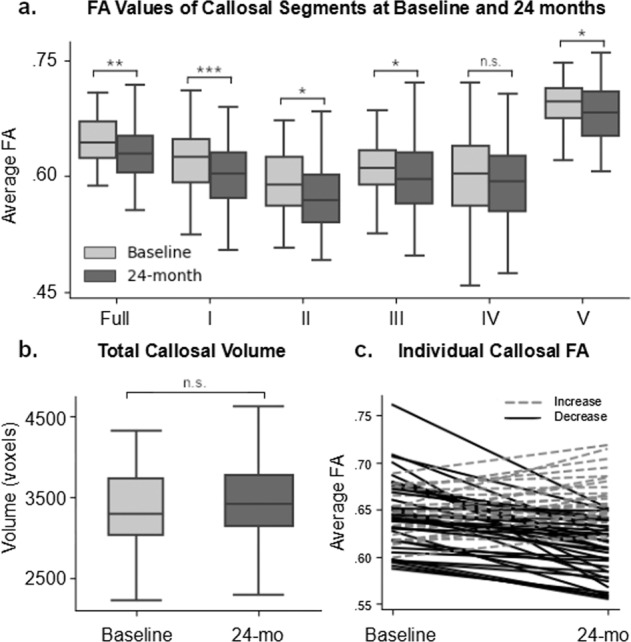
Longitudinal analysis of individually-based CC ROI’s. **a** This bar chart shows the FA values for the full CC ROI and each callosal segment at the baseline (light gray) and 24-month (dark gray) follow-up time-points. Error bars displayed are 95% confidence intervals. **b** Averaged callosal volume indexed in number of voxels at the baseline and 24-month follow-up time-points. Voxels are 2 mm^3^. **c** Individual FA changes for the full corpus callosum from baseline to 24-month follow-up. Each line is a subject, with dashed and solid lines indicating increases and decreases, respectively. Annotations: *p < 0.05; **p < 01; ***p < 0.001; n.s. not significant.

To examine the microstructural integrity of CC more precisely and to avoid normalization and group-averaging problems, we extracted FA values from the hand-drawn ROIs for each individual in their native space at both baseline and 24 months. Figure 2a shows the average FA for each callosal segment at the two time-points. 36 out of 61 subjects (59%) showed FA decline over two years, while the rest showed little or no change or a slight increase (Fig. 2c). The average decrease in FA in the full CC ROI was 0.043 (SD = 0.032) and the average increase in FA was 0.019 (SD = 0.016). A within-subject repeated measure analysis of variance (ANOVA) revealed significant main effects of time [F(1, 60) = 8.74, *p* < 0.005] and callosal segment [F(4, 60) = 190.24, *p* < 0.0001], but their interaction did not reach statistical significance [F(4, 60) = 2.22, *p* = 0.07]. Post-hoc *t* tests revealed that all callosal segments, except IV, showed a significant decrease in FA over the 2 years (*t’s*(60) *>* 2.00, *p’s* < 0.05, corrected for multiple comparisons), while the differences in FA change across the callosal segments were very small or negligible [F(4, 300) <1, *p* = 0.5]. To rule out potential volume related confounds (Fig. 2b), we further confirmed that there was no significant difference between the full callosal ROI volume between the baseline and 24-month time-point, although the volumes of the premotor and motor sections appeared larger at the 24-month time-point.

**Fig. 3:**
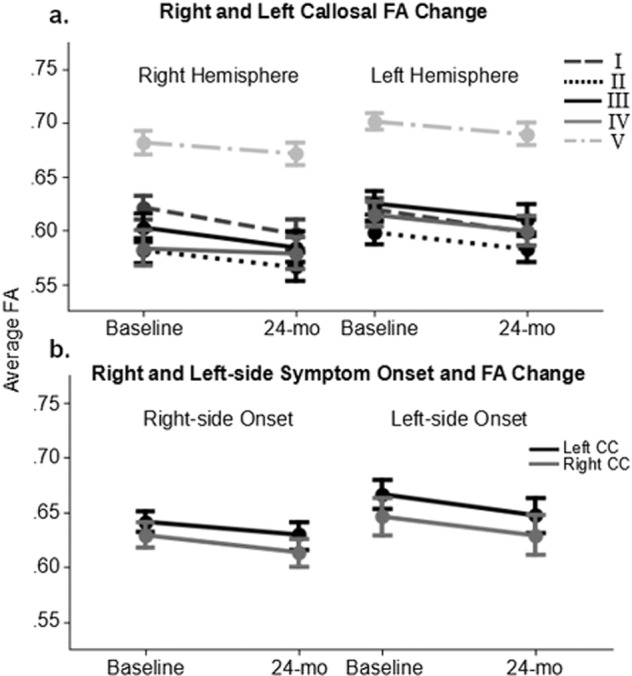
Lateralized and onset-based callosal microstructural changes. **a** Change in FA of the left and the right hemisphere of the corpus callosum over two years. Each line indicates a different callosal segment. **b** Changes in FA in the left and right hemispheres of the corpus callosum for subjects with left and right symptom onset. I = prefrontal section; II = premotor section; III = motor section; IV = somatosensory section; V = temporal-parietal-occipital section.

We further examined whether the FA changes were lateralized according to the side of motor symptom onset by dividing the patients into right-onset and left-onset groups (Fig. 3b). The ANCOVA showed that FA decline was not significantly different by onset side [F(1, 57) <1]. We conducted a time by hemisphere repeated measures ANOVA for each onset group to test whether the side of onset was associated with lateralized callosal FA decline (Fig. 3b). We found significant main effects of time [right-onset: F(1, 32) = 4.65, *p* < 0.05; left-onset: F(1, 25) = 4.21, *p* < 0.05] and hemisphere [right-onset: F(1, 32) = 15.07, *p* < 0.0005; left-onset: F(1, 25) = 18.07, *p* < 0.01] for both groups, but not the time by hemisphere interactions [right-onset: F(1, 32) <1; left-onset: F(1, 25) = 2.64, *p* = 0.12]. It is worth mentioning that the callosal FA was lower in the right hemisphere compared to the left hemisphere at both baseline [*t*(60) = 4.44, *p* < 0.0001] and 24-month [*t*(60) = 11.15, *p* < 0.0001] time-points (Fig. 3a).

In sum, there was a significant decline in FA across the two-year period in most callosal segments, albeit to various extents. This effect was evident in 59% of the sample and was consistent for both right and left-onset individuals, but there were no clear lateralization effects in association with the side of symptom onset.

### Correlation between FA and motor symptoms

**Fig. 4:**
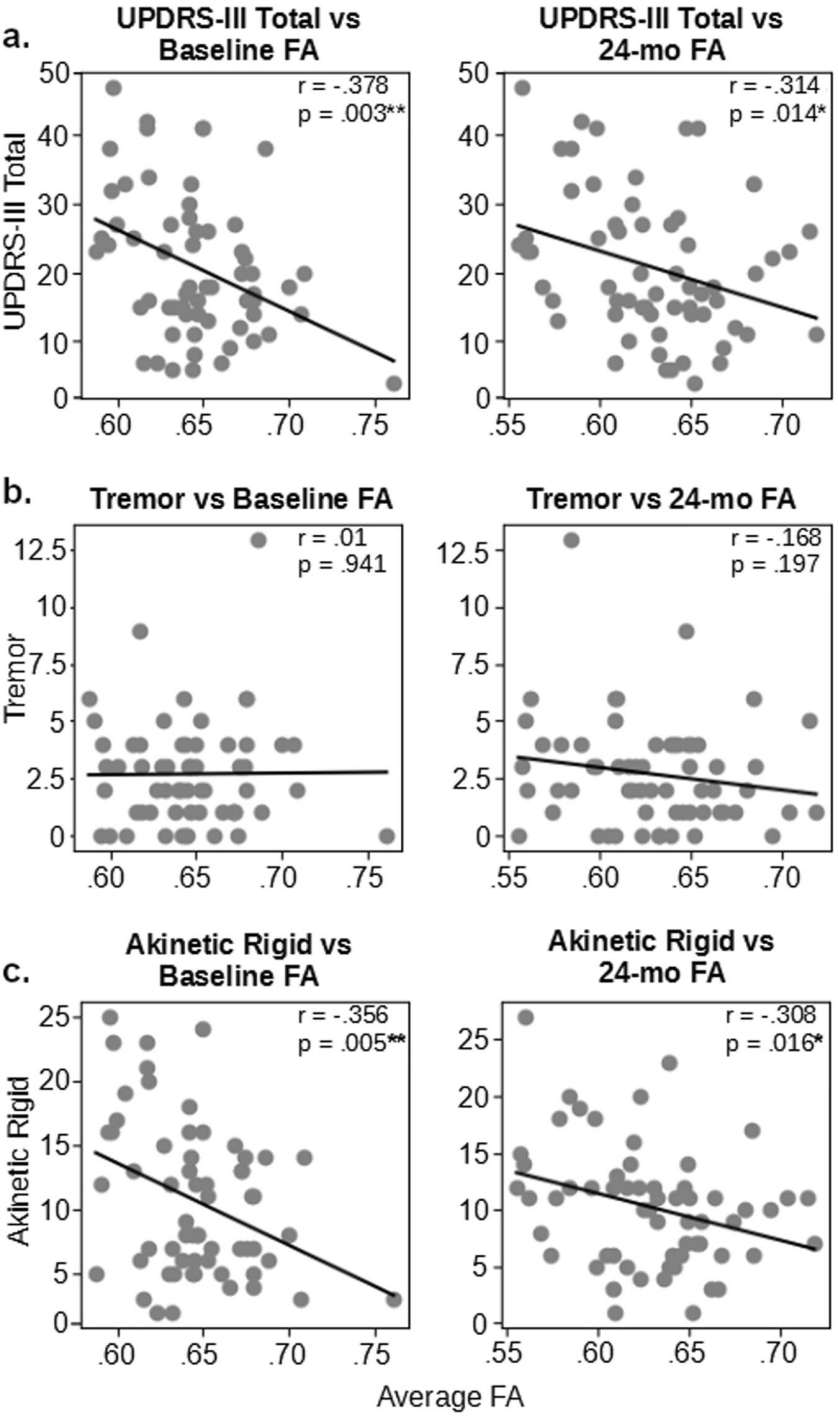
UPDRS-III total and akinetic-rigid scores are associated with FA of the full corpus callosum at both time points. Scatter plots in the left show across-subject correlation between motor symptoms and baseline CC FA and those in the right show the correlation with 24-months FA. **a** The bivariate relationship between baseline and 24-month UPDRS-III with baseline CC FA. **b** The bivariate relationship between baseline and 24-month tremor UPDRS-III subscores with baseline CC FA. **c** The bivariate relationship between baseline and 24-month akinetic-rigid UPDRS-III subscores with baseline CC FA. Annotations: *p < 0.05; **p < 1.

To examine the relationship between microstructural integrity at both time points and baseline Parkinsonian motor symptom severity, we conducted multiple-regression analyses. Microstructural integrity was measured by FA levels at either time-point, and motor symptom severity levels were extracted from UPDRS-III. Age, scan site, gender, and MoCA score were included in all of the models as covariates. For baseline FA, the model was approaching significance for UPDRS-III total [F(5, 55) = 2.33, *p* = 0.055, *R*^*2*^ = 0.17] and reached significance for AR severity [F(5, 55) = 2.40, *p* = 0.049, *R*^*2*^ = 0.18]. For 24-month FA, the model was significant for UPDRS-III total [F(5, 55) = 2.55, *p* < 0.05, *R*^*2*^ = 0.19] and AR severity [F(5, 55) = 3.23, *p* < 0.05, *R*^*2*^ = 0.23], with both the UPDRS-III and AR models having significant FA coefficients. In contrast, the model for tremor did not reach significance for either time-points [baseline: F(5, 55) = 1.58, *p* = 0.18, *R*^*2*^ = 0.13]; 24-month: F(5, 55) = 1.66, *p* = 0.16, *R*^*2*^ = 0.13]. These different effects are shown in Fig. 4.

To further explore whether the relationship between FA and motor symptoms varies across the CC at each time-point, additional regression analyses were conducted for each subdivision. For baseline UPDRS-III total, the model for the prefrontal section was significant [F(5, 55) = 2.94, *p* < 0.05, *R*^*2*^ = 0.21], with a significant FA coefficient. For baseline AR, the prefrontal section [F(5, 55) = 2.75, *p* < 0.05, *R*^*2*^ = 0.20] and somatosensory section [F(5, 55) = 2.46, *p* < 0.05, *R*^*2*^ = 0.18] reached significance. For 24-month UPDRS-III total, the prefrontal [F(5, 55) = 2.46, *p* < 0.05, *R*^*2*^ = 0.18], motor [F(5, 55) = 2.53, *p* < 0.05, *R*^*2*^ = 0.19], and temporal-parietal-occipital sections [F(5, 55) = 2.72, *p* < 0.05, *R*^*2*^ = 0.20] reached significance, with significant FA coefficients. For AR, all models reached significance (*p* < 0.05, *R*^*2*^ = 0.18–0.24), with the exception of the somatosensory section [F(5, 55) = 2.30, *p* = 0.057, *R*^*2*^ = 0.17]; FA coefficients of the prefrontal, motor, and temporal-parietal-occipital sections reached significance, with the prefrontal section showing the strongest effect (*R*^*2*^ = 0.24). Again, none of the models reached significance for tremor (*p’s* > 0.05, *R*^*2*^ = 0.13–0.14).

In sum, while the different sections of the CC were related with motor symptom severity to various degrees, the microstructural integrity of the anterior callosal sections showed a consistent relationship with motor symptom severity, particularly with the prefrontal FA decline more closely relating to AR symptom severity across subjects at both time-points.

### Exploratory voxel-wise correlates with CC microstructural changes

**Fig. 5:**
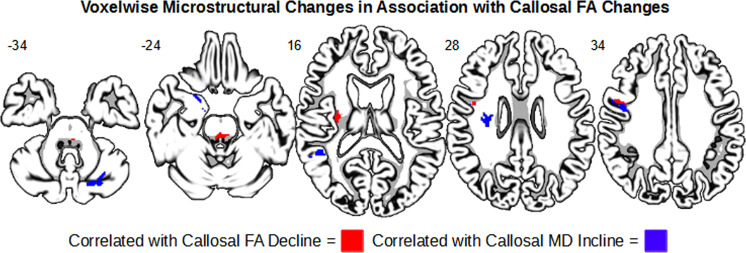
Whole-brain regression maps showing changes in callosal FA in association with widespread microstructure decline. Red indicates areas where callosal FA decline was associated with FA decrease. Blue indicates areas where callosal FA decline was associated with MD increase.

Finally, we explored whether FA-related degradations in the CC were associated with microstructural degradation in the rest of the brain over the 24-month period. There were widespread significant changes in both FA and MD associated with callosal FA change, as shown in Fig. 5, including many regions implicated in PD, such as the cerebellum, thalamus, and hippocampus.

## Discussion

We examined the pattern of longitudinal microstructural degradation of the CC in early-stage PD using the PPMI sample with DTI data at multiple time-points. In the voxel-wise, whole-brain analysis of FA, both the genu and splenium of the CC showed significant decline over 24 months. However, analyses using the individually defined ROIs revealed that four of five callosal segments showed a significant FA decline over two years. Such microstructural changes were strongest in the most anterior portions of the CC, the prefrontal (section I) and premotor subsections (section II). We also found significantly lower FA in the right CC compared to the left CC, while both right and left-onset subjects showed a similar pattern of callosal microstructural decline over time. Further, across subjects, low FA at both baseline and 24 months was closely associated with more severe AR symptoms and not so with tremor. An exploratory analysis showed that the 24-month decline in callosal microstructural integrity was associated with widespread microstructural decline in the rest of the brain. Taken together, these findings suggest that there is significant microstructural decline over two years in early-stage, drug-naive PD, which may serve as a marker for AR severity.

The findings from our whole-brain and ROI analyses indicate that multiple subdivisions of the CC degrade to a certain extent within the first two years in individuals newly diagnosed with PD. In particular, the areas of the CC that exhibited a significant decline were the prefrontal, premotor, motor, and temporal-parietal-occipital subsections, leaving the somatosensory subsection as the only potentially unperturbed cortical connection. These findings corroborate with past literature in this field. While there are only a few longitudinal DTI studies on PD, some investigators reported significant callosal changes over time using voxel-wise approaches^10,26,27,33^. Recently, Wu and colleagues took a similar approach to ours and created CC ROIs and divided them into the same five functional segments, based on Hofer and Frahm’s 2006 paper^26^. They showed in a more advanced PD sample (mean H&Y score of 2.5), each CC segment, except the temporal-parietal-occipital subsection, showed FA decline and MD increase over a 2-year time period. However, their method differed from ours in that they incorporated automated ROI creation, in which each individual’s brain was normalized to a template for group-level analysis, rather than creating ROIs in each individual’s native space. See supplementary materials for potential misalignment issues caused by normalization (Figure S1). Nevertheless, we observed similar changes.

We suggest that the individually-based ROI approach may allow for more precise measurement of callosal microstructural changes in PD including early stages and potentially prodromal stages that cross-sectional and voxel-wise approaches would not have the sensitivity and resolution to detect. As discussed in the introduction, previous whole-brain analyses have been inconsistent about the exact anatomical changes, with many reporting change in either the genu^6,11,20^ or splenium^21–24^, but not both, and few have observed changes in multiple sections of the CC while others found no significant effects in the same CC regions^34,35^. Similarly, our initial voxel-wise approach replicates the genu and splenium findings, but it clearly did not capture the full effect shown by the more intricate ROI analysis. Importantly, our ROI findings also reveal that the degree to which the Parkinsonian motor symptomatology relates to different subsection decline is separable.

Our findings do not suggest that there is a strong difference in rate of decline between the CC subregions in early PD. It is possible that the moderate sample size was still underpowered or the sample we studied was too early to show more diverging microstructural differences across the different callosal segments. A longer scale longitudinal study into a later stage is required to examine if the anterior CC degrades faster than the posterior CC in PD over time. Nonetheless, our ROI findings extend the previous literature by showing detectable FA decline in multiple CC functional subdivisions within the first 24 months of newly diagnosed individuals with PD. Our exploratory analysis showed that the 24-month microstructural change in the CC is also associated with widespread microstructural changes in brain regions implicated in PD, including the cerebellum, thalamus, and hippocampus. Future studies of callosal decline in PD should further examine how the different sections’ degradation is associated with changes in brain function. As each section connects with different cortical areas across the two hemispheres, their microstructural decline presumably would have different functional consequences.

Our findings also reveal that baseline motor symptom severity, especially AR severity, is linked to lower baseline and 24-month follow-up callosal microstructural integrity across subjects. There is limited data to this date relating callosal microstructural integrity to motor symptoms in PD, with a few other studies reporting an association between callosal FA and motor symptoms. For instance, three different studies have found that higher UPDRS-III total is related to lower FA and higher MD values^26,36,37^. Wen and colleagues found that in tremor dominant groups, UPDRS-III total was correlated with lower FA in the genu^36^. Additionally, using tractography to partition the CC, Galantucci and colleagues found a negative association between UPDRS-III total and FA in the genu, body, and splenium of the CC in an early-stage cohort, as well as a positive association between UPDRS-III and MD in the body and the splenium^37^. Wu and colleagues showed that heightened MD in the temporal-parietal-occipital subsection of the CC is related to higher UPDRS-III scores^26^. Our study replicated and extended some of these previous findings in a larger early-stage sample. In particular, we found that the microstructural integrity of the anterior portions of CC, which showed the most decline in FA over time, were more consistently linked to worse motor symptoms across subjects at both baseline and 24 months.

Our findings further suggest that AR scores, but not tremor scores, were more closely linked to FA levels in these frontal callosal sections. While this trend was observed for multiple callosal segments, the prefrontal section of the CC seemed to be especially sensitive to microstructural damage at this stage of the disease in relation to AR symptom, linking callosal microstructural integrity and AR severity in early-stage PD. There has been related research in the past. For instance, Chan and colleagues showed that a significantly higher rate of falling in patients at later stages of PD was related to a lower FA in the body of the CC^20^. Since the PPMI cohort used in our analysis were drug-naive, de novo PD patients at baseline, our findings suggest that microstructural changes in the CC may play a role in early signs of AR and disease progression. Indeed, we have previously found that AR, but not tremor severity, was related to altered functional connectivity between the cortical motor areas in a similar cohort of early-stage PD subjects^38^.

The finding of a closer link between AR and the microstructural integrity of the prefrontal and motor subsections of CC is intriguing because of their particular connection patterns with the cortex^14^. Given the anatomy, it is possible that these callosal sections may play a crucial role in connecting the frontal and parietal motor areas across the hemispheres, and disruption to these connections may worsen akinetic-rigid symptoms, as previous research has shown that interhemispheric communication between the motor and supplementary cortices are crucial for initiation and planning of voluntary movement^39^. Further, the current findings of these callosoal microstructure changes provide an anatomical underpinning to our previous findings of worse AR symptoms in association with altered functional connectivity between the pre-SMA and paracentral lobule, and between the anterior portions of the superior parietal lobule^38^. These findings together suggest that alterations of callosal microstructure may underlie altered functional connectivity, and these changes may have implications for understanding PD subtype development.

These findings are especially interesting when taken in context of our exploratory whole-brain regression analysis, where we found that decline in callosal FA predicted widespread microstructural decline. Some of these affected areas included the primary motor cortex and the cerebellum, both closely implicated in Parkinsonian motor dysfunctions^40,41^. Given the anatomy of CC, it is not surprising that its microstructural changes may allude to a larger anatomical pattern of microstructural degradation. Thus, even if the effect on these brain regions in early PD may not be advanced enough to be fully encapsulated using DTI, their association with the CC may point to future degradation and functional decline. Taken together, callosal microstructural degradation may accompany widespread microstructural degradation and have particular implications in the heterogeneity of symptomatology across individuals with PD.

Our study has some limitations, including the lack of a control group. Without healthy controls for comparison, we were unable to directly verify if the callosal degradation observed in this early-stage PD sample is accelerated compared to healthy aging. To mitigate this issue, age was included as a confounding variable in the statistical models, and our findings of microstructural changes over time seem to be beyond simple age differences. It is also worth mentioning that past studies using a cohort from the PPMI database found that over a one-year follow-up, PD subjects had significant decline in the CC compared to control subjects^33^. A similar limitation is that we did not compare our PD sample to other neurodegenerative disorders. Indeed, CC microstructural alterations have been found in other neurodegenerative disorders including Alzheimer’s disease (e.g., Teipel et al.)^42^ and Huntington’s disease (e.g., Di Paola et al.)^43^, as well as healthy aging (e.g., Weis, Kimbacher, Wenger, & Neuhold)^44^. However, our goal of this study is not to claim that callosal degradation is unique to PD. Rather, we utilized individually-based ROI analysis to systematically show how microstructural alteration in the whole CC and its subdivisions relates to Parkinsonian symptomatology and functional outcomes.

Another limitation with our study is that while the hand-drawn ROIs allowed us to avoid introducing partial volume effects from the nearby highly isotropic ventricular space, it was difficult to distinguish the boundary separating the lateral body of callosal white matter and cerebral white matter. Since the cerebral white matter tends to have lower FA than callosal FA^45^, this may affect the FA measures for some CC segments. Nonetheless, this problem is common and a greater problem in the typical group-level, voxel-wise measures, with the whole-brain and auto-segmentation methods ignoring the problem altogether and likely introducing unwanted partial volume effects due to the nature of the methodology.

In conclusion, we used individually drawn ROIs comprising the full 3D volume of the CC and measured FA changes over a two-year longitudinal scale in early-stage, drug-naive PD, with a moderate sample size. Significant microstructural decline was evident in four of five callosal segments, even in this early stage, with two thirds of the sample showing some level of decline. The anterior CC, particularly the prefrontal section, appears to show the most microstructural decline over 24 months, as well as demonstrating a consistent association with AR severity across subjects at both time-points. These data suggest that FA changes in the CC may be a viable marker of AR and disease progression in early-stage PD.

## Methods

### PD sample and DTI dataset

All behavioral and neuroimaging data from newly diagnosed PD patients was obtained from the Parkinson’s Progressive Markers Initiative (PPMI) database (www.ppmiinfo.org/data). For up-to-date information on the study, visit www.ppmi-info.org. PPMI is a multi-site longitudinal program, funded by the Michael J. Fox Foundation for Parkinson’s Research and funding partners (www.ppmiinfo.org/fundingpartners). Data samples from 162 participants were downloaded from the database on 1 November 2019. DTI data were from seven scan sites, with 103 participants having a baseline time-point, 141 participants having a 12-month time-point, 130 participants having a 24-month time-point, and among them, 77 participants having both baseline and 24-month data. We only included participants who were classified as level I or II on the Hoehn & Yahr scale for PD symptom severity at baseline. Note that none of the participants were using antipsychotic or antiepileptic medication at the time of scanning. Overall cognitive ability was measured using the Montreal Cognitive Assessment (MoCA), and Parkinsonian motor symptom severity was measured using part III of the Unified Parkinson’s Disease Rating Scale (UPDRS-III).

Anatomical T1 MPRAGE images were acquired on a Siemens 3 T TIM Trio scanner, with repetition time (TR) = 2300 ms, echo time (TE) = 2.98 ms, flip angle = 9°, matrix = 240 × 256, field of view (FOV) = 256 mm, voxel size 1 × 1 × 1 mm^3^, 176 sagittal slices with slice thickness = 1 mm. Diffusion MR sequences were acquired with a Siemens 3 T TIM Trio scanner, with TR = 900 ms, TE = 88 ms, flip angle = 90°, voxel size 2 × 2 × 2 mm^3^, 72 sagittal slices with slice thickness = 2 mm. There were 64 gradient directions with a *b*-value = 1000 s/mm^2^, and one reference non-gradient volume (*b* = 0 s/mm^2^). To minimize noise confounds, each DWI volume was visually inspected to screen for artifacts, movement, or anatomical abnormalities that might alter the data.

### Image quality control, DTI image preprocessing and CC ROIs

We took multiple steps to ensure image quality and longitudinal data reliability, including visual inspection of all images, noise removal and correction, and analysis validation. First, if visual inspection showed a volume had visible artifacts that corrupted the image quality, such as loss of signal or lines due to motion, that volume was removed from the dataset using the 3dCalc tool in Analysis of Functional Images (AFNI, https://afni.nimh.nih.gov/)^46^. As the PD population often had motor disturbances, there were a considerable amount of volumes that were removed. Individuals with more than 25 volumes of DTI data (39%) removed due to artifacts were excluded from the further analysis, leaving 94 baseline datasets, 136 12-month datasets, and 123 24-month datasets. As a result, 61 participants that had baseline, 12-month, and 24-month datasets were included in the final analysis. Table 2 shows the demographic data of these 61 individuals with DTI at multiple time-points.

**Table 2:** Demographic data of all subjects.

	Baseline	24 month	Stats (*t* value, *p* value)
Age	61.6 ± 9.2	-	-
Gender	38 M, 23 F	-	-
Affected side (right/left/symmetrical)	33/26/2	-	-
Hoehn & Yahr	1.51 ± .5	1.75 ± .67	2.96, 0.004*
MoCA	27.1 ± 2.3	26.7 ± 3.16	0.54, 0.29
UPDRS-III total	20.8 ± 10.4	23.5 ± 14.0	1.66, 0.051
Akinetic rigid	10.7 ± 5.9	12.4 ± 7.7	2.31, 0.01*
Tremor	2.7 ± 2.4	2.7 ± 3.0	−1.78, 0.96
LEDD	-	250.6 ± 203.6	-

MoCA Montreal Cognitive Assessment, UPDRS-III Total Unified Parkinson’s Disease Rating Scale part III total, Akinetic Rigid summed UPDRS scores for akinetic rigid symptoms, Tremor summed UPDRS scores for tremor symptoms, LEDD mean levodopa equivalent daily dose.

For each of the 61 subjects, we applied standard preprocessing steps, including motion correction, eddy-current correction, and rigid-body registration to a common space, and created a diffusion matrix, using the Tolerably Obsessive Registration and Tensor Optimization Indolent Software Ensemble (TORTOISE, https://tortoise.nibib.nih.gov/tortoise)^47^. In comparison to other common DTI pipelines, TORTOISE is less automated and takes steps to ensure the user is involved and quality checking at each step of the processing pipeline. This ensures the data are high quality, and no artifacts are affecting the results. FA maps and directionally encoded color (DEC) maps were created using AFNI for all three time-points for each subject. For the group-level analyses, the FA images of each individual were coregistrated and normalized to the PD MNI template^48^ using Statistical Parametric Mapping 12 (SPM12, https://www.fil.ion.ucl.ac.uk/spm/)^49^. Spatial smoothing was applied with a full width half maximum of 6 mm.

**Fig. 6:**
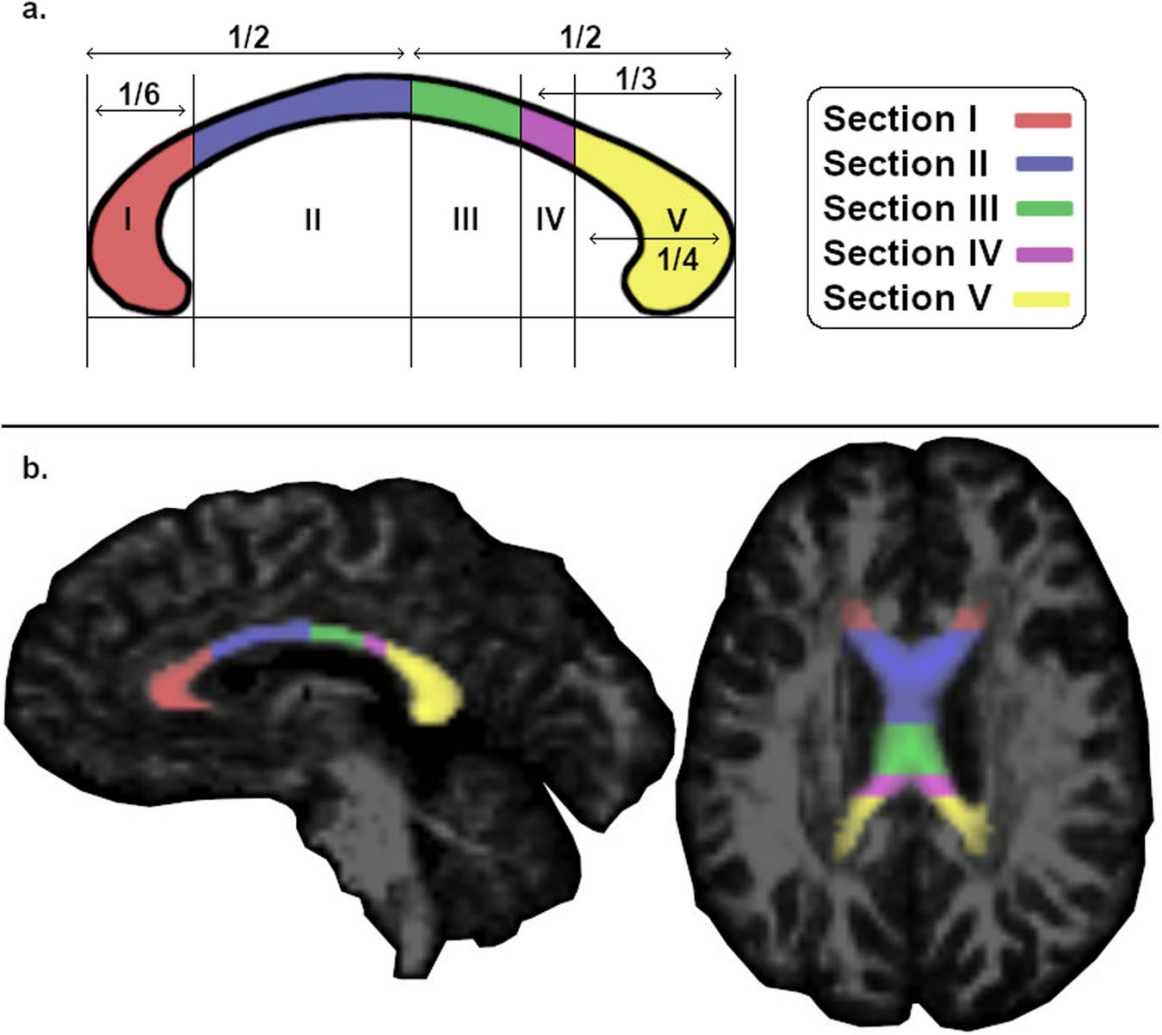
Segmentation of the corpus callosum. **a** Corpus callosum segmentation scheme as in the study by Hofer and Frahm (2006). **b** An example of a fully segmented hand-drawn corpus callosum ROI in sagittal and axial view. The five callosal segments are illustrated in different colors. I = prefrontal section; II = premotor section; III = motor section; IV = somatosensory section; V = temporal-parietal-occipital section.

The DEC images were used as a guide to ensure that primarily callosal volume was included in the hand-drawn ROI for each individual. More specifically, 3D ROIs were drawn for each participant’s CC, starting on the mid-sagittal plane and extending laterally until callosal fibers were no longer distinguishable, using ITK-Snap (www.itksnap.org). Every individual’s CC at baseline and 24-month was drawn and visually inspected by three trained lab personnel, who were blinded to the time-point. In order to control for drawing error, we employed median absolute deviation (MAD) outlier detection from AFNI on each ROI. This removed potential outlier voxels within the ROI and ensured quality of these manually drawn images, as the lateral edges of the CC are difficult to distinguish from the nearby white matter. We then separated each of the CC ROIs into five distinct functional segments and used them as masks for each individual participant’s native space FA images. These five segments originate from Hofer and Frahm’s parcellation scheme based on DTI tractography of the human CC^14^ (see Fig. 6). The five segments are topographically organized and have differential cortical connections from anterior to posterior as follows: prefrontal (section I), premotor (section II), motor (section III), somatosensory (section IV), and temporal-parietal-occipital (section V). Using the FSLmath function in FSL, these segments were created automatically for each individual’s CC ROI, and visually inspected for quality control. To track callosal changes using the individually defined ROIs, average FA values for each segment of the CC were extracted for each subject at both baseline and 24-month time-points.

Besides visual inspection of the hand-drawn ROIs by multiple raters and evaluating volume differences across time-points, we took an additional step to assure that the ROI results were not confounded by drawing errors. Each hand-drawn ROI was normalized to the PD MNI template and paired *t* tests were used to compare FA at the baseline and 24-month time-points. Using the same baseline and 24-month normalized ROIs, we thresholded the images using the top 90, 75, and 50 percent of voxels and applied paired *t* tests again to test for potential fitting errors and voxel number dependence. Indeed, the callosal FA effect stayed consistent across the different pairs of ROIs.

As PPMI is a database that utilizes data from multiple scan sites, we further examined variability across sites. Since there are still debates on how to address noise and reliability issues from multiple scan sites^50,51^, we attempted to address this issue with the following steps. With the overall DWI image quality, we conducted a one-way ANOVA testing for the main effect of scan site on whole-brain signal-to-noise ratio (tSNR) in the diffusion images. To determine scan-re-scan reliability, we conducted a one-way ANOVA testing for the main effect of time-point on tSNR. Neither test showed differences that were statistically significant (F’s < 1). Importantly, previous studies have demonstrated that ROI analyses show better reliability across longitudinal scans^50^. Following past longitudinal DTI literature on reliability testing^52^, we conducted two two-way mixed effects model intra-class correlation (ICC) with absolute agreement using FA values extracted from the John Hopkins University MNI (JHU)^53^ white matter atlas’s CC ROI. Both the baseline to 12-month (ICC = 0.975, *p* < 0.0001), and the 12-month to 24-month (ICC = 0.985, *p* < 0.0001) analyses were significant, suggesting the data were reliable across time-points. We also included scan site as a categorical covariate in all multiple-regression analyses. Hence, we believe that the variance attributed from multiple scan sites is negligible in our results.

### Statistical analysis

For the group-level, voxel-wise whole-brain analysis, we used SPM12 (https://www.fil.ion.ucl.ac.uk/spm/software/spm12/) to conduct one-way repeated measures ANOVAs on the subjects with DTI data at baseline, 12-month, and 24-month time-points to determine which gray matter and white matter regions showed FA and MD changes across the three time-points. Group maps were thresholded at *p* < 0.001 and *k* > 9, with clusters deemed significant if they survived FDR correction of *p* < 0.05.

For the ROI analyses, we focused on FA because FA is shown to be a more reliable and reproducible measure of microstructural integrity in white matter areas of dense axon fibers compared to MD^54^. Using the FA values extracted from the hand-drawn masks from each individual, we conducted a two-way repeated measures ANOVA to test for the main effects of time, callosal segment, and their interaction. We conducted additional paired *t* tests to test for potential volumetric differences across time for the whole CC ROI, as well as for each segment.

To examine whether the CC showed lateralized FA changes, we divided the sample into two separate groups: left symptom onset (*n* = 26) and right symptom onset (*n* = 33). Subjects who had bilateral symptom onset (*n* = 2) were excluded from these analyses. A three factor analysis of covariance (ANCOVA) was applied to test for the main effects of time, hemisphere, and side of symptom onset on FA of the full CC, with age included as a covariate. We further used 2 × 2 ANOVA to test whether within-subject changes in FA across time showed different hemispheric effects for the left and right-onset groups.

To examine how microstructural decline in the different CC subdivisions relates to symptom severity in early PD, we applied multiple-regression analyses to examine the relationship between motor symptoms and callosal FA at baseline and a 24-month follow-up across subjects. Three separate models were conducted: one predicting overall motor symptom severity (UPDRS-III total score), one predicting akinetic-rigid sub-score (AR; UPDRS-III AR Sum), and one predicting tremor sub-score (UPDRS-III Tremor Sum)^55^. Similarly, we examined the association between callosal FA and general cognitive impairment. Cognitive ability was indexed by the total MoCA score^56^. For both MoCA and UPDRS-III total scores, the baseline measurement was used for each subject in the respective analyses. Age, gender, and scan site were included as confounding variables in all regression models.

Finally, to explore the potential impact of early CC degradation on the rest of the brain, we examined how changes in FA over 24 months in the full callosal ROI correlate with voxel-wise FA and MD changes in the rest of the brain. First, baseline versus 24-month microstructural alteration FA and MD maps were created using the ImCalc function, using the FA and MD images of all 61 individuals. For FA, the difference maps were created by subtracting each individual’s 24-month time-point images from their baseline images. For MD, the difference maps were created by subtracting each individual’s baseline images from their 24-month images. That is, positive values in both difference maps indicate microstructural decline over time. Second, the ROI callosal regressor was calculated by subtracting the average 24-month full CC FA from the baseline CC FA for each individual subject. Third, the FA/MD difference maps together with the callosal regressor were used in the SPM12 voxel-wise whole-brain multiple-regression analysis, with age, gender, and years of education as covariates. The resulting regression maps were thresholded at *p* < 0.001 and *k* > 9, with clusters deemed significant if they survived FDR correction of *p* < 0.05.

### Standard protocol approvals, registrations, and patient consents

This study was approved by the institutional review board at all PPMI sites, and written informed consent was obtained from all participants in this study at the original sites of research.

## Supplementary information

Supplementary Figure 1

## Data Availability

Anonymized data will be made available by request from any qualified investigator by emailing one of the corresponding authors. All original datasets, including imaging and behavioral data, are available through PPMI (ppmi-info.org), and any investigators interested may apply for access to the data through the PPMI portal.

## Code availability

Code for data analysis and separating a hand-drawn CC ROI into its corresponding functional sections will be made available by request from any qualified investigator by emailing one of the corresponding authors.
