# Supplementary material for: Longitudinal corpus callosum microstructural decline in early-stage Parkinson’s disease in association with akinetic-rigid symptom severity
Source: NPJ Parkinsons Dis. 2022 Aug 29;8:108. doi: 10.1038/s41531-022-00372-1 (PMC9424284; doi:10.1038/s41531-022-00372-1)
Supplement: Supplementary file 1 — Supplementary Figure 1 [file 41531_2022_372_MOESM1_ESM.pdf]

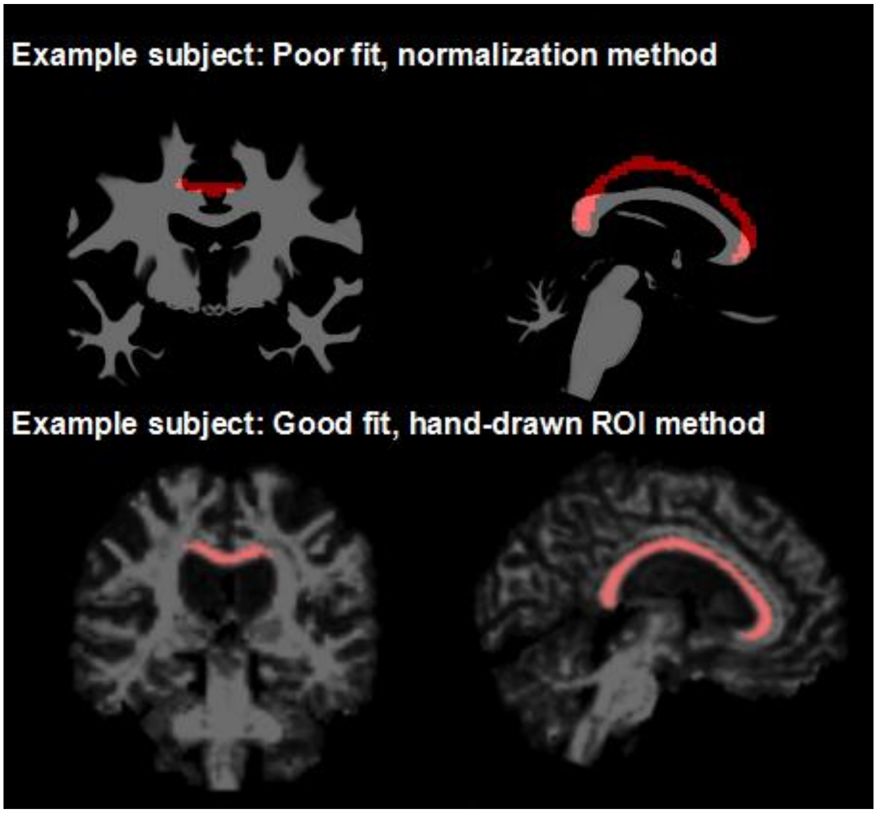

Supplemental Figure 1 – Misalignment issues caused by normalization. (Top) An example of a poorly fitting ROI in MNI template space as a result of spatial normalization. (Bottom) The same subject's hand-drawn ROI in the individual's native space.
